# Integrated genomic analysis and functional characterisation of novel oncogenes in ovarian cancer

**DOI:** 10.1186/1897-4287-10-S2-A81

**Published:** 2012-04-12

**Authors:** SJ Davis, KJ Simpson, IG Campbell, KL Gorringe

**Affiliations:** 1 VBCRC Cancer Genetics Laboratory, Peter MacCallum Cancer Centre, St. Andrew’s Pl., East Melbourne, Victoria, Australia; 2 Department of Pathology, The University of Melbourne, Parkville, Victoria, Australia; 3 Victorian Centre for Functional Genomics, Peter MacCallum Cancer Centre, St. Andrew’s Pl., East Melbourne, Victoria, Australia

## 

Ovarian cancer is associated with the highest mortality rate of all gynaecologic malignancies, and is identified as the sixth most common cause of cancer death for Australian women**.** Genomic copy number amplification is a hallmark of oncogene associated tumour development and progression. The targeted increase in copy number of such chromosomal regions has a significant impact upon gene expression that imparts selective advantages on cancer cells. **These over-expressed genes are attractive therapeutic targets,** particularly as increased gene expression within the cancer genome is often associated with ‘oncogene addiction’.

To identify amplicon targets we have adopted a methodology that combines genomic copy number and expression data with RNA interference (RNAi) to identify amplified genes that are functionally relevant. According to our analysis of SNP array data over 46 Mb of the genome was amplified as a high level in 10% or more tumours, encompassing ~300 genes. This set of candidate genes will be functionally assessed *in vitro* using a boutique siRNA library to interogate phenotypic alterations in proliferation and morphology. We have utilized SNP6.0 array data from 39 ovarian cell lines to identify 18 cell lines that recapitulate the gene amplifications observed in clinical specimens, which form the basis of this study.

Data obtained from the first cell lines examined indicates that despite the different genetic backgrounds of the cell lines used, there is overlap in the genes that cause significant alterations to cellular proliferation. Furthermore, a number of genes not previously associated with ovarian cancer have been shown to impart functional effects directly associated with genomic amplification. This data provides evidence that the high-throughput nature of this functional screen will rapidly identify candidate genes that may then be streamlined to a validation phase. The identification and validation of such genes is critical to their translation as potential therapeutic targets.

